# Psychiatric hospital utilisation following lithium discontinuation in patients with bipolar I or II disorder: A mirror-image study based on the lisie retrospective cohort

**DOI:** 10.1192/j.eurpsy.2021.238

**Published:** 2021-08-13

**Authors:** L. Öhlund, M. Ott, M. Bergqvist, S. Oja, R. Lundqvist, M. Sandlund, E. Salander Renberg, U. Werneke

**Affiliations:** 1 Sunderby Research Unit, Department Of Clinical Sciences, Division Of Psychiatry, Umeå University, Umeå, Sweden; 2 Department Of Public Health And Clinical Medicine, Division Of Medicine, Umeå University, Umeå, Sweden; 3 Department Of Psychiatry, Piteå Älvdals hospital, Piteå, Sweden; 4 Sunderby Hospital, Department of Psychiatry, Luleå, Sweden; 5 Department Of Public Health And Clinical Medicine, Sunderby Research Unit, Umeå University, Umeå, Sweden; 6 Department Of Clinical Sciences, Division Of Psychiatry, Umeå University, Umeå, Sweden

**Keywords:** bipolar disorder, lithium, Admission, mood stabiliser

## Abstract

**Introduction:**

Evidence for lithium as a maintenance treatment for bipolar disorder type II remains limited since most treatment-prevention studies focus on bipolar disorder type I or do not distinguish between types of bipolar disorder.

**Objectives:**

To compare the impact of lithium discontinuation on hospital utilisation in patients with bipolar disorder type I or schizoaffective disorder and patients with bipolar disorder type II or other bipolar disorder.

**Methods:**

Mirror-image study, examining hospital utilisation within two years before and after lithium discontinuation as part of LiSIE, a retrospective cohort study into effects and side-effects of lithium for the maintenance treatment of bipolar disorder as compared to other mood stabilisers.

**Results:**

For the whole sample, the number of admissions increased from 86 to 185 admissions after lithium discontinuation, with the mean number of admissions/patient/review period doubling from 0.44 to 0.95 (p < 0.001). The number of bed days increased from 2218 to 4240, with the mean number of bed days/patient/review period doubling from 11 to 22 (p = 0.025). This increase in admissions and bed days was exclusively attributable to patients with bipolar disorder type I or schizoaffective disorder.

**Conclusions:**

Our findings suggest that due to a higher relapse risk in patients with bipolar disorder type I or schizoaffective disorder there is a need to apply a higher threshold for discontinuing lithium than for patients with bipolar disorder type II or other bipolar disorder.

**Disclosure:**

Michael Ott has been a scientific advisory board member of Astra Zeneca Sweden, Ursula Werneke has received funding for educational activities on behalf of Norrbotten Region (Masterclass Psychiatry Programme 2014–2018 and EAPM 2016, Luleå, Sweden): Astra

